# EcoCurrentNet an integrated DNN-CatBoost model for predicting optoelectronic material performance under varying conditions

**DOI:** 10.1038/s41598-025-14510-1

**Published:** 2025-11-24

**Authors:** Sun Xiaoying

**Affiliations:** Department of Electrical and Computer Engineering, Sungkyunkwan University, Sunwon, Korea

**Keywords:** Other photonics, Electrical and electronic engineering

## Abstract

Simulating the performance of optoelectronic materials under complex and variable environmental conditions in laboratory settings presents a significant challenge, as laboratory environments often fail to accurately replicate real-world conditions. This limitation hinders the reliability of performance assessments for optoelectronic materials in practical applications. To address this limitation, this research introduces EcoCurrentNet, an innovative model that integrates deep neural networks (DNN) with convolutional layers and residual blocks, combined with a CatBoost regression layer, to effectively capture spatial and nonlinear dependencies. The convolutional component learns the interactions between material features and 12 environmental variables, while residual blocks enhance training stability and gradient flow across deeper layers. A global average pooling and fully connected layer compress the learned features before they are passed to the CatBoost regressor, which iteratively refines the final output. The model is designed based on principles of thermodynamics and material science, particularly the complex relationships between material properties and external factors such as temperature, pressure, and light intensity, which can be described by physical laws governing material behavior. The model achieves a remarkable R^2^ score of 99.68%, demonstrating its capability to provide accurate assessments of material behavior beyond controlled laboratory conditions. This hybrid architecture illustrates the potential of combining deep residual learning and gradient boosting for modeling complex physical systems, offering a more reliable and efficient approach to material design and paving the way for future innovations in the field.

## Introduction

Accurate prediction of optoelectronic material performance under varying environmental conditions is essential for the advancement of photonic and electronic applications. Traditional methods, such as physics-based models and many machine learning (ML) approaches, often fall short in capturing the complex, nonlinear interactions between environmental factors and material properties. The interdependent nature of factors such as temperature, humidity, light intensity, and pressure makes it challenging for conventional models to incorporate these variables effectively. Consequently, these traditional models often exhibit limitations in both predictive accuracy and generalization under diverse conditions^1,2^.

Recent studies have highlighted significant predictive gaps in models that rely solely on single-variable or physics-based approaches. These models typically fail to account for the compounded effects of multiple environmental factors, leading to inaccurate predictions in practical scenarios^3,4^. For instance, temperature can significantly influence the bandgap energy of semiconductors, thereby altering their optical absorption spectra. Materials like Gallium Arsenide (GaAs) exhibit red-shifting of their absorption edge as temperature increases, which directly impacts the performance of devices such as photodetectors or solar cells. This type of unexpected behavior under varying temperature conditions illustrates the importance of modeling such interdependencies in optoelectronic systems.

To address these challenges, this research introduces EcoCurrentNet, a novel hybrid model that combines the strengths of deep neural networks (DNNs) for feature extraction with the boosting power of CatBoost for enhanced generalization. EcoCurrentNet is designed to capture the intricate relationships between material properties and environmental factors. The deep feature extraction capabilities of DNNs allow the model to identify subtle patterns within optoelectronic data, while CatBoost’s boost technique improves generalization, particularly when traditional machine learning models may struggle to perform optimally^5–7^. Recent reviews have further emphasized the urgency of modeling environmental interactions in optoelectronic systems, such as studies on stability in hybrid materials^8^, molecular-level strategies for device optimization^9^, and nanoscale frameworks for environmental sensing^10^. These findings underscore the need for predictive tools like EcoCurrentNet that can integrate environmental complexity into practical material simulations.

EcoCurrentNet represents a significant advancement in the field of optoelectronic material analysis. By integrating twelve environmental factors such as temperature, light intensity, humidity, and pressure EcoCurrentNet provides a more comprehensive simulation of real-world conditions. Although this research does not involve experimental testing on physical materials, the theoretical analysis and model validation using five representative optoelectronic materials-Silicon (Si), Gallium Arsenide (GaAs), Indium Phosphide (InP), Germanium (Ge), and Zinc Oxide (ZnO) demonstrate its exceptional performance under various material conditions. These theoretical validations offer valuable insights into EcoCurrentNet’s potential for use across diverse environmental scenarios and provide a theoretical foundation for future optoelectronic material design.

This research makes an important theoretical contribution to the development of predictive models for optoelectronic materials. By capturing the complex dependencies between material behavior and environmental variables, EcoCurrentNet offers a robust framework for future material design. Although the study is based on simulated data, its results provide valuable guidance for the development of smart materials, offering a new perspective for the design of next-generation optoelectronic and electronic devices. The integration of deep learning and gradient boosting techniques allows EcoCurrentNet to overcome the limitations of conventional approaches, ensuring accurate and adaptable performance prediction under complex real-world conditions^11–15^.

## Results

This research evaluated the performance of the integrated deep learning model, **EcoCurrentNet**, against several traditional machine learning (ML) methods, including the Bagging Regressor, Random Forest, Decision Tree, Ridge Regression, Bayesian Ridge, Linear Regression, Support Vector Regression (SVR), and K-Nearest Neighbors Regressor (KNeighbors Regressor). The performance of these models, particularly in terms of R^2^ scores, is summarized in Table 1.

**Table 1:** R^2^ Scores of different models.

Model	R^2^
EcoCurrentNet	0.996526
Bagging regressor	0.989263
Random forest	0.988846
Decision tree	0.978542
Ridge regression	0.968972
Bayesian ridge	0.968969
Linear regression	0.968963
Support vector regression	0.928274
KNeighbors regressor	0.770642

This research evaluated the performance of the integrated deep learning model, **EcoCurrentNet**, against several traditional machine learning (ML) methods, including Bagging Regressor, Random Forest, Decision Tree, Ridge Regression, Bayesian Ridge, Linear Regression, Support Vector Regression (SVR), and K-Nearest Neighbors Regressor (KNeighbors Regressor). EcoCurrentNet achieved the highest performance with an R^2^ score of 0.996526, indicating an excellent fit to the data and significantly outperforming all other models. Bagging Regressor (0.989263), Random Forest (0.988846), and Decision Tree (0.978542) also exhibited strong predictive capabilities, but their performance was inferior to that of EcoCurrentNet. These models were effective in capturing nonlinear relationships and feature interactions; however, they demonstrated limitations in handling complex, multidimensional variables. Models such as Ridge Regression (0.968972), Bayesian Ridge (0.968969), Linear Regression (0.968963), and Support Vector Regression (SVR, 0.928274) showed good predictive performance but were less adept at modeling the complex nonlinear relationships between environmental variables and material properties. As noted by Sun et al.^16^, “Traditional regression models often exhibit substantial biases when addressing complex nonlinear relationships”, and Mahmoud et al.^17^ further highlighted that “Deep learning methods outperform traditional regression models in extracting latent patterns from high-dimensional, complex data”.

The K-Nearest Neighbors Regressor (KNeighbors Regressor) showed the lowest performance with an R^2^ score of 0.770642. This is attributed to its sensitivity to noise and local features, especially in high-dimensional spaces where the relationships between variables are intricate and the sample size is limited. As Zhang et al.^18^ stated, “K-Nearest Neighbors regression is particularly susceptible to noise in high-dimensional spaces, especially when the sample size is small, and struggles to capture global patterns”.

EcoCurrentNet’s superior performance can be attributed to its hybrid architecture, which integrates the strengths of deep neural networks (DNNs) for feature extraction and CatBoost for enhanced generalization. This combined approach enables EcoCurrentNet to effectively capture nonlinear dependencies and interactions among the multiple variables influencing optoelectronic material performance. Sun et al. ^16^ observed that “Deep neural networks are capable of automatically extracting features from complex data, while CatBoost’s gradient boosting framework further enhances the model’s generalization ability”, and Zhang et al. ^18^ noted that “The integration of DNN and CatBoost allows EcoCurrentNet to handle high-dimensional and complex nonlinear relationships effectively”. This integrated approach results in significant improvements in predicting the behavior of optoelectronic materials under varying conditions.

This integrated approach results in significant improvements in predicting the behavior of optoelectronic materials under varying conditions.

**Training and inference time.** In addition to accuracy, the computational efficiency of EcoCurrentNet was also assessed. On a standard workstation equipped with an Intel i7 CPU and 32GB RAM, the training process for EcoCurrentNet (with 10,000 samples and 100 epochs) took approximately 20 seconds, while inference for a single sample took less than 2 milliseconds. Compared to traditional machine learning models, EcoCurrentNet’s training time was moderately higher due to the DNN component; however, its inference time remained competitive, especially when deployed in batch mode. These results indicate that EcoCurrentNet is not only accurate but also computationally efficient, making it suitable for practical applications involving large-scale environmental data.

## Discussion

This research evaluated the performance of the integrated deep learning model, **EcoCurrentNet**, against several traditional machine learning (ML) models, including Ridge Regression, Bayesian Ridge Regression, Lasso Regression, Linear Regression, K-Neighbors Regression, and Support Vector Regression (SVR)^14,19,20^. With an R^2^ value of 0.996526, EcoCurrentNet shows a significant advantage in capturing the complex nonlinear relationships present in the dataset, outperforming traditional models.

### Performance comparison analysis

The high accuracy of EcoCurrentNet emphasizes the advantages of combining deep learning and gradient boosting techniques in predicting material performance. While traditional models like Ridge Regression, Bayesian Ridge Regression, and Linear Regression can effectively capture linear dependencies, their performance is significantly limited when faced with complex interactions^21,22^. These models typically assume linear relationships between features, making them inflexible in adapting to potential nonlinear features and interactions in the dataset.

In high-dimensional spaces, the K-Nearest Neighbors Regressor (KNeighbors Regressor) faces significant challenges, reflecting its limitations in handling optoelectronic material performance data. The performance of KNeighbors Regressor is influenced by its sensitivity to the choice of distance metrics and the number of neighbors; when the number of features exceeds the number of samples, KNeighbors Regressor is prone to overfitting, leading to insufficient generalization capabilities^1,23^. This limitation underscores the importance of selecting an appropriate algorithm when building predictive models.

In contrast, EcoCurrentNet successfully captures the complex nonlinear relationships in optoelectronic material performance data by combining the strengths of DNN and CatBoost. DNN excels in feature extraction and nonlinear mapping, identifying complex patterns in the data, while CatBoost has unique advantages in handling categorical features and achieving efficient gradient boosting^2,3^. This integrated approach allows EcoCurrentNet to generalize well across different environmental conditions and material properties, effectively capturing subtle dependencies.

### Importance of the EcoCurrentNet integrated model

The integrated approach of EcoCurrentNet combines DNN, CatBoost, and residual blocks, forming a powerful predictive model. DNN performs exceptionally in feature extraction and nonlinear mapping, enabling it to recognize complex patterns and potential nonlinear relationships in the data. However, traditional DNN may encounter challenges in dealing with categorical features and high-dimensional data, which is the strength of CatBoost^14^. CatBoost is specifically designed to handle categorical features using efficient splitting strategies to minimize overfitting, thereby improving the model’s predictive accuracy.

The introduction of residual blocks further enhances the performance of EcoCurrentNet. These residual structures facilitate smoother gradient propagation within the network through skip connections, alleviating the common issue of vanishing gradients encountered in training deep networks^3^. This design enables the model to learn complex features more deeply while retaining the ability to capture lower-level features. Thus, EcoCurrentNet achieves excellent generalization across different environmental conditions and material properties, effectively capturing subtle dependencies.

This combination not only improves the model’s accuracy in predicting optoelectronic material performance but also illustrates how to integrate different types of learning methods to enhance predictive modeling capabilities, especially in scenarios with limited data. The design concept of this hybrid model provides an innovative solution in the field of materials science, particularly in exploring new material combinations and environmental scenarios, showcasing significant application potential.

### Consistency with theoretical expectations

To verify that the predictions made by EcoCurrentNet are consistent with established optoelectronic theory, a simplified test case was designed. In this scenario, only three variables light intensity, electric field strength, and temperature were varied, while all other factors were held constant. According to fundamental physical laws, the current in optoelectronic devices is approximately proportional to the product of charge carrier mobility, electric field, and carrier concentration.

The model output showed a nearly linear increase in predicted current as light intensity and electric field strength increased, which matches expectations from the classical drift-diffusion model. Temperature variations initially led to a rise in current due to enhanced carrier mobility, followed by a saturation point again consistent with theoretical observations in semiconductor materials.

These results demonstrate that EcoCurrentNet not only captures complex multi-variable interactions but also aligns with well-known physical behaviors under controlled, idealized conditions.

### Significance and future applications

These results highlight the potential of EcoCurrentNet in optimizing optoelectronic materials, providing a tool for exploring new material combinations and environmental scenarios without the need for extensive physical testing. By accurately simulating material performance, this research can offer valuable insights, accelerate the design process, and reduce experimental costs. The integration of machine learning into materials science, as demonstrated in recent studies, underscores its transformative impact. For instance, Stein et al. illustrated how machine learning models could predict optical spectra and facilitate the discovery of materials with desired optical properties^24^. Similarly, the work by Chong et al. discussed the broad advancements in applying machine learning techniques to model and predict material behaviors effectively, showcasing the potential for expanding frameworks like EcoCurrentNet^25^.

Future research could expand the framework of this model by incorporating additional material characteristics or environmental factors, enabling broader applications in the field of materials science. Integrating these techniques aligns with the emerging trends in leveraging machine learning for deeper, more accurate simulations and enhanced generalization capabilities in complex material systems^24,25^.

These results emphasize the potential of EcoCurrentNet in optimizing optoelectronic materials, providing tools for exploring new material combinations and environmental scenarios, thereby filling the gap in the field of materials science. By accurately simulating material performance, this research can provide valuable insights, accelerate the design process, and lower experimental costs. Future research may expand the model’s framework by incorporating more material characteristics or environmental factors, achieving wider applications in materials science.

## Methods

The system setup was specifically chosen to ensure compatibility and efficient execution of deep learning tasks, with a particular focus on managing dependencies and ensuring reproducibility. Computational resources were adequate for the models used in this study; however, larger datasets and more complex models may require additional hardware capabilities. The code and all required software dependencies for this work are available for replication and transparency.

### Experimental environment

The experiments were conducted in a Python-based environment with the following system and software configurations as shown in Table 2.

**Table 2:** System and environment details used for the experiments.

Component	Details
Operating system	Windows 10 (64-bit)
Python version	3.9.19
Python executable	c:$\backslash$Users$\backslash$Monet$\backslash$anaconda3$\backslash$envs$\backslash$EcoCurrentNet$\backslash$python.exe
Platform	Windows-10-10.0.19045-SP0
Architecture	64-bit (WindowsPE)
Core Libraries	NumPy (v1.26.4)
	Pandas (v2.2.1)
	TensorFlow (v2.7.0)
	Keras (v2.7.0)
	Scikit-learn (v1.5.2)
	XGBoost (v2.1.2)
	LightGBM (v4.5.0)
	CatBoost (v1.2.7)
	Matplotlib (v3.8.3)
	Seaborn (v0.13.2)
Hardware	Intel processor
Environment	Anaconda Virtual Environment for managing dependencies

### Data generation and physical rationale

**Fig. 1:**
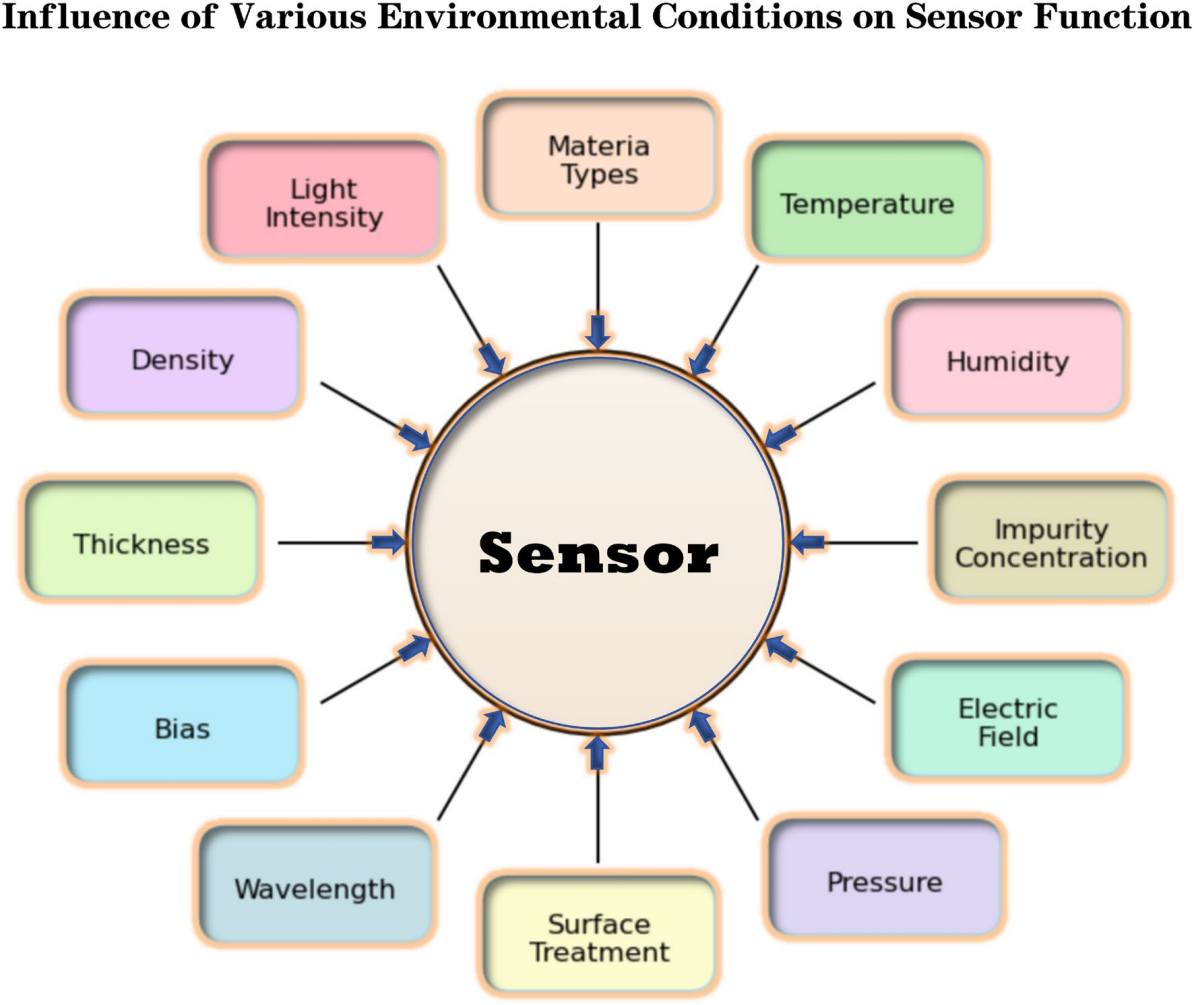
Depicts the environmental factors, such as temperature and humidity, that influence the performance of optoelectronic sensors.

In this study, this research designed and implemented a specialized data generator to generate suitable performance data for optoelectronic materials. The core of this generator is to simulate the responses of optoelectronic materials under different conditions through randomized parameters. The main variables that influence the performance of optoelectronic materials were identified, including light intensity, temperature, material type, humidity, wavelength, pressure, thickness, impurity concentration, bias voltage, surface treatment, electric field strength, and density (see Fig. 1)^1–3^.

The data generator class, OptoelectronicDataGenerator, accepts the number of samples n_samples as a parameter during initialization and sets a fixed reverse saturation current $I_s$. Material properties are defined as a dictionary that includes five different optoelectronic materials along with their corresponding response coefficients: Silicon, Gallium Arsenide, Indium Phosphide, Germanium, and Zinc Oxide^1,14,26^. The response coefficients for each material are set relative to a baseline material to facilitate weighting in subsequent current calculations.

The data generation process is completed through the generate_data method. This research uses the NumPy library to generate the specified number of samples, which include: light intensity (ranging from 0 to 800,000 Lux), temperature (ranging from −20 to 80 °C), material type (integer values from 0 to 4 representing different materials), humidity (ranging from 0 to 100%), wavelength (ranging from 400 to 700 nm), pressure (ranging from 900 to 1100 hPa), thickness (ranging from 0 to 10 $\upmu$m), impurity concentration (ranging from 0 to 2%), bias voltage (ranging from −5 to 5 V), surface treatment (0 for none, 1 for treated), electric field strength (ranging from 0 to 1000 V/m), and material density (ranging from 1 to 11 g/cm³)^20,27,28^. The detailed content of the generated feature dataset X can be found in Table 3. The corresponding current values y are calculated through the calculate_current method.

**Fig. 2:**
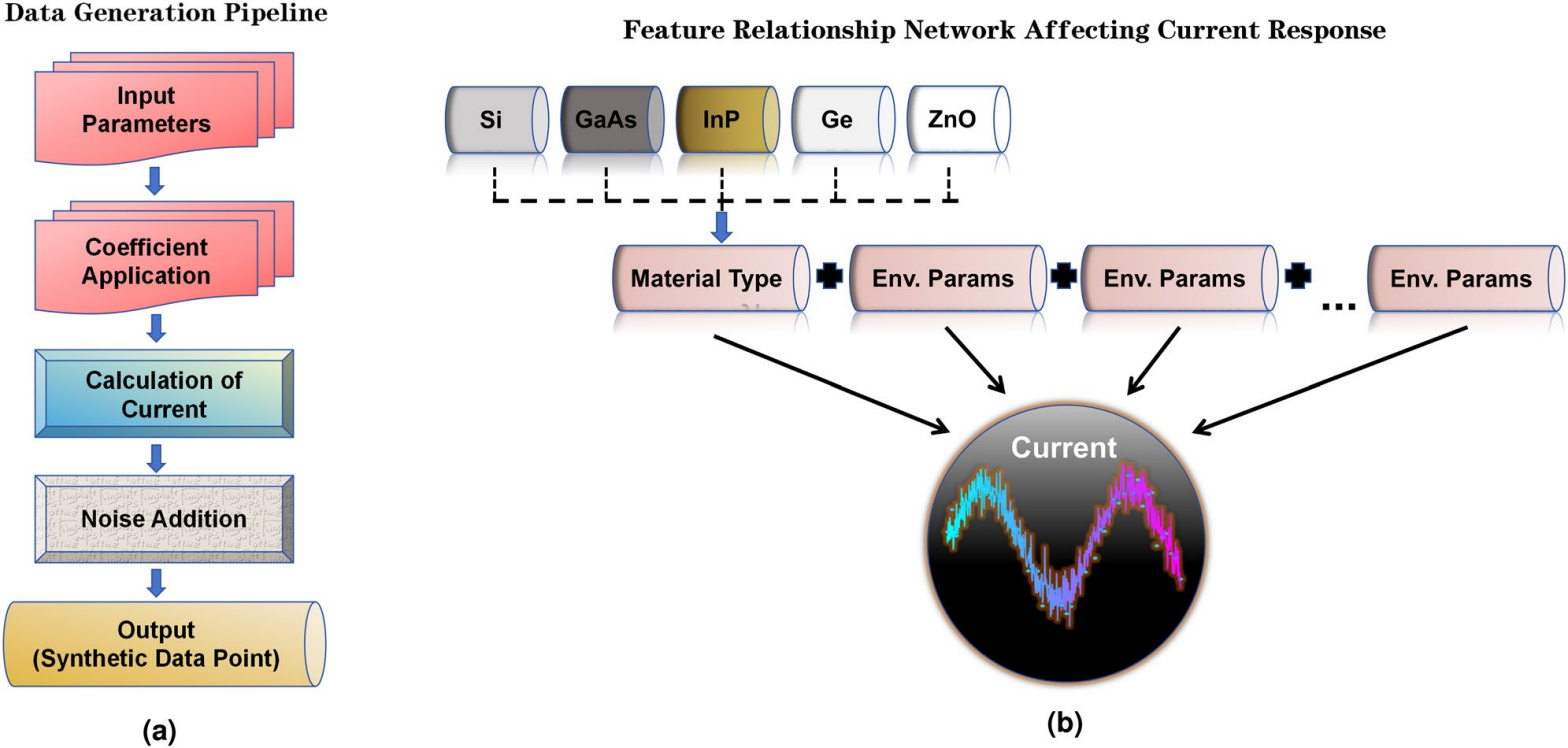
Overview of the data generation process and feature relationships. (**a**) Flow diagram illustrating the steps from input parameters to synthetic dataset creation. (**b**) Flowchart depicting the interactions and influence of input parameters on optoelectronic material performance.

The current calculation formula incorporates all influencing factors and adds random noise to simulate uncertainties in actual measurements (see Fig. 2)^1,14,26^. Additionally, the analysis of significant features highlights how the predefined material coefficients weight the responses of different materials, thereby reflecting the performance differences of various materials under specific conditions^20,27,28^. The formula for the calculated current is as follows:

1 $$\begin{aligned} I = & \left( L \cdot C_m + T \cdot C_T + H \cdot C_H + W \cdot C_W + P \cdot C_P + T_h \cdot C_{th} + I_m \cdot C_I \right.\\ & \left. + B \cdot C_B + S \cdot C_S + E \cdot C_E + D \cdot C_D + I_s \right) + \epsilon \end{aligned}$$

Where:$I$: calculated current, $L$: light intensity, $T$: temperature, $H$: humidity, $W$: wavelength, $P$: pressure, $T_h$: thickness, $I_m$: impurity concentration, $B$: bias voltage, $S$: surface treatment, $E$: electric field strength, $D$: material density, $I_s$: reverse saturation current, $\epsilon$: random noise, $C_m, C_T, C_H, C_W, C_P, C_{th}$ $C_I, C_B, C_S, C_E, C_D$ : material coefficients

**Table 3:** Parameter ranges and rationale.

Parameter	Range	Rationale
Material Type	Silicon, GaAs, InP, Ge, ZnO	Includes common materials used in optoelectronic applications.
Light Intensity	0 to 800,000 Lux	Covers a wide range for optimal performance in various conditions.
Temperature	−20 to 80 °C	Ensures testing across extreme environmental conditions.
Humidity	0 to 100%	Examines effects of humidity on material performance.
Wavelength	400 to 700 nm	Represents the visible light spectrum for electronic response.
Pressure	900 to 1,100 hPa	Pressure conditions typical in various applications.
Thickness	0 to 10 $\upmu$m	Allows for variations in material applications.
Impurity concentration	0% to 2%	Low levels of impurities are critical for material efficiency.
Bias	−5 to 5 V	Varies bias to analyze material responses under different conditions.
Surface treatment	Treated (1) or untreated (0)	Surface treatments can significantly alter material properties.
Electric field strength	0 to 1,000 V/m	Electric field effects critical for performance analysis.
Material density	1 to 11 g/cm^3^	Density affects material properties and device efficiency.

In the current calculation, the noise $\epsilon$ is randomly generated based on a normal distribution to simulate uncertainties in actual measurements. Specifically, the generation of noise can be represented by the following formula:

2 $$\begin{aligned} \epsilon \sim \mathcal {N}(0, \sigma ^2) \end{aligned}$$

*Where:* $\sigma$ is the standard deviation of the noise. In this way, the generated current values consider not only the weighted sum of various influencing factors but also incorporate randomness caused by measurement errors and environmental changes.

To ensure the validity and usability of the generated data, this research performed preprocessing on the data. In the preprocess_data method, this research first standardized the feature data to improve the efficiency and accuracy of model training, as suggested by recent works in the field of optoelectronics and machine learning optimization techniques^26,28^. Subsequently, the data was divided into training and validation sets to support the following model training and evaluation.

**Fig. 3:**
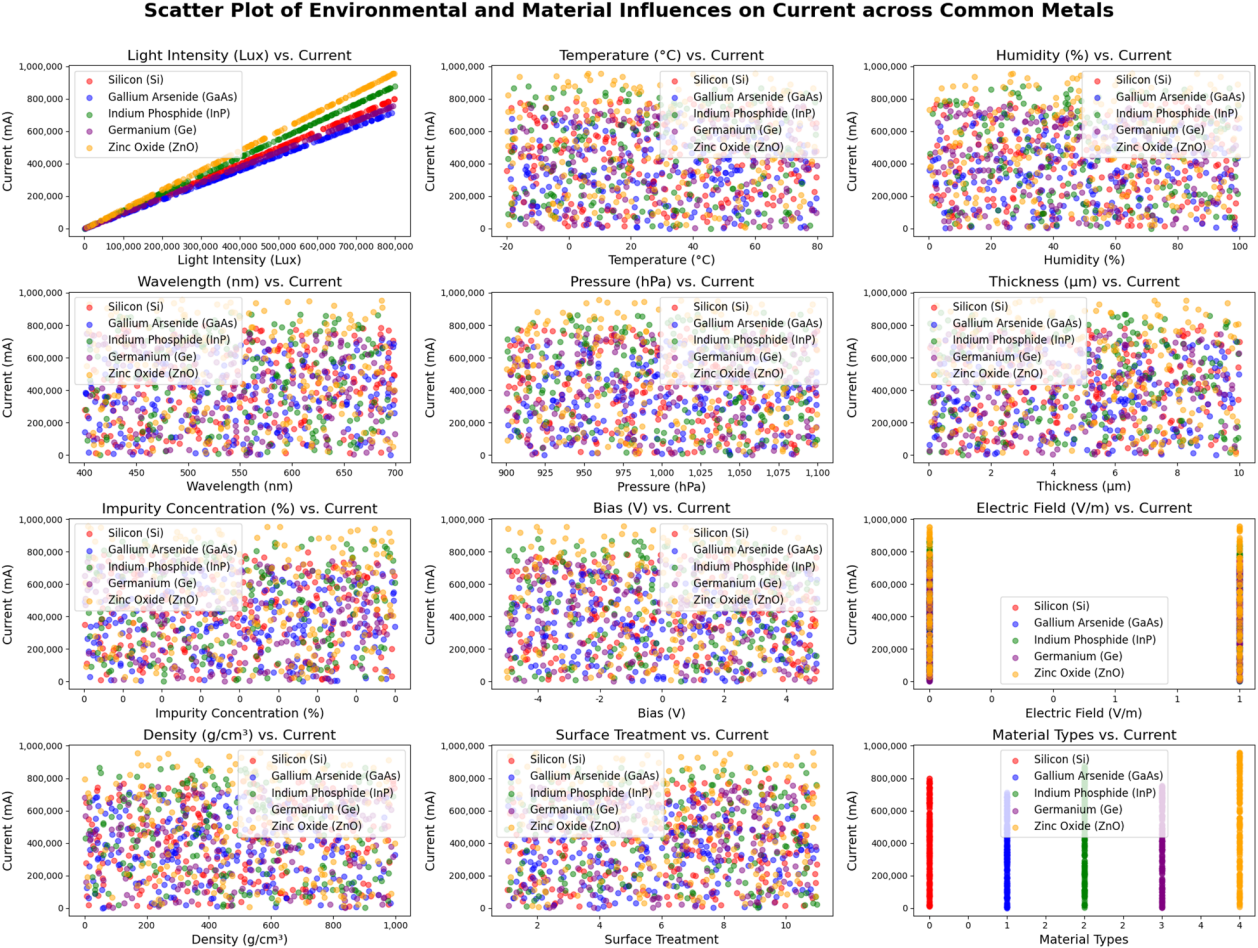
Analysis of significant features impacting the performance of optoelectronic materials, highlighting critical parameters for future research.

Through this process, this research successfully generated a comprehensive dataset of optoelectronic material performance considering multiple variables, providing the necessary input for subsequent deep learning models. Specifically, Fig. 3 illustrates the analysis of key features impacting optoelectronic material performance, highlighting significant parameters critical for future research. These key features play an essential role in the model, helping identify whichphysical and environmental factors have the most considerable influence on material performance.

In this study, the current values were synthesized using a model inspired by well-established optoelectronic principles, rather than relying on strictly physical models. The data generation process considers both additive and multiplicative relationships between environmental factors and material properties. This approach is partially based on the drift-diffusion model and extended photoelectric current equations. For instance, photoelectric current is typically modeled as proportional to carrier mobility ($\mu$), electric field ($E$), and carrier concentration ($n$):

3 $$\begin{aligned} I \propto q \cdot \mu \cdot E \cdot n \end{aligned}$$

where $\mu$ is the carrier mobility, $E$ is the electric field, and $n$ is the carrier concentration, all of which are affected by temperature, impurity concentration, and pressure. This relationship offers a foundational understanding of how material behavior is influenced by various environmental conditions.

Moreover, environmental variables like humidity and surface treatment have been reported to significantly influence the charge transport behavior in optoelectronic materials. These relationships are taken into account in our model, where the current is expressed as a weighted sum of twelve environmental factors, reflecting the nonlinear interactions between the inputs (material properties and environmental conditions) and the resulting output (current). While the model does not directly simulate a full physical process, it closely approximates known photoelectric behaviors, offering a reliable theoretical testing platform for evaluating model performance under complex environmental conditions^29–32^.

While Fig. 3 indicates that some factors (e.g., surface treatment, material density) show weaker correlations with the current, these factors are deliberately retained in the model. In actual optoelectronic systems, the interactions between factors are typically nonlinear and context-dependent. Variables that seem weak in isolation may become significant under specific combinations of inputs. By including these twelve environmental factors, the model maintains flexibility and robustness, enabling it to learn complex dependencies. Furthermore, the feature selection mechanism in CatBoost automatically assigns lower importance to irrelevant variables during training, thus preventing unnecessary model complexity.

**Fig. 4:**
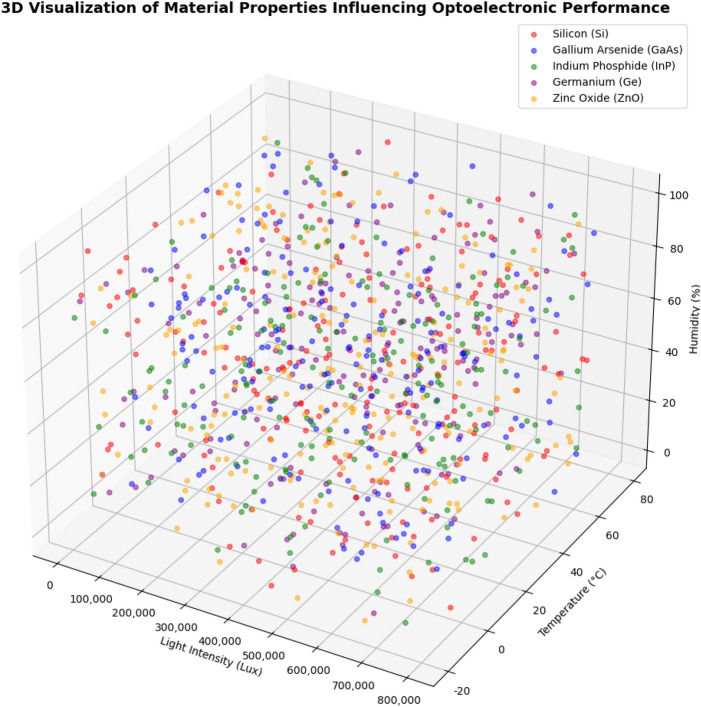
A 3D visualization of key material properties, illustrating trends and correlations among different characteristics affecting optoelectronic performance.

Additionally, Fig. 4 presents a 3D scatter plot of material properties, visualizing the trends and correlations between different characteristics that affect optoelectronic performance. This plot helps reveal how the material properties interact with each other, influencing overall performance, thereby providing an intuitive understanding for further optimization of material design.

These visualizations offer valuable insights into the process of generating current and deepen our understanding of how different features interrelate, helping to better comprehend the interactions and impacts between various factors on material performance.

## Model selection

To enhance the predictive capabilities of EcoCurrentNet for optoelectronic material responses, this research systematically evaluated several model architectures and optimization strategies. This research designed and compared five versions of EcoCurrentNet (EcoCurrentNet_V1 to EcoCurrentNet_V5), each incorporating distinct configurations to explore varying levels of model complexity and depth. This iterative design approach enabled this research to identify the architecture best suited to capturing the nuanced relationships in the data. Additionally, this research examined the performance impact of six optimizers-RMSprop, Adam, Adagrad, Adamax, Nadam, and Ftrl-on each model variant. These trials helped determine the optimal combination of model architecture and optimizer, ultimately leading to the selection of RMSprop in combination with EcoCurrentNet_V4, supported by CatBoost as an auxiliary model, to achieve the highest R^2^ score and predictive robustness Table 4.

**Table 4:** R^2^ scores for different models and optimizers.

Model version	Optimizer	R^2^
EcoCurrentNet_V4	RMSprop	0.9847
EcoCurrentNet_V4	Adam	0.9800
EcoCurrentNet_V4	Adamax	0.9743
EcoCurrentNet_V3	Nadam	0.8066
EcoCurrentNet_V3	Adam	0.7635

**Fig. 5:**
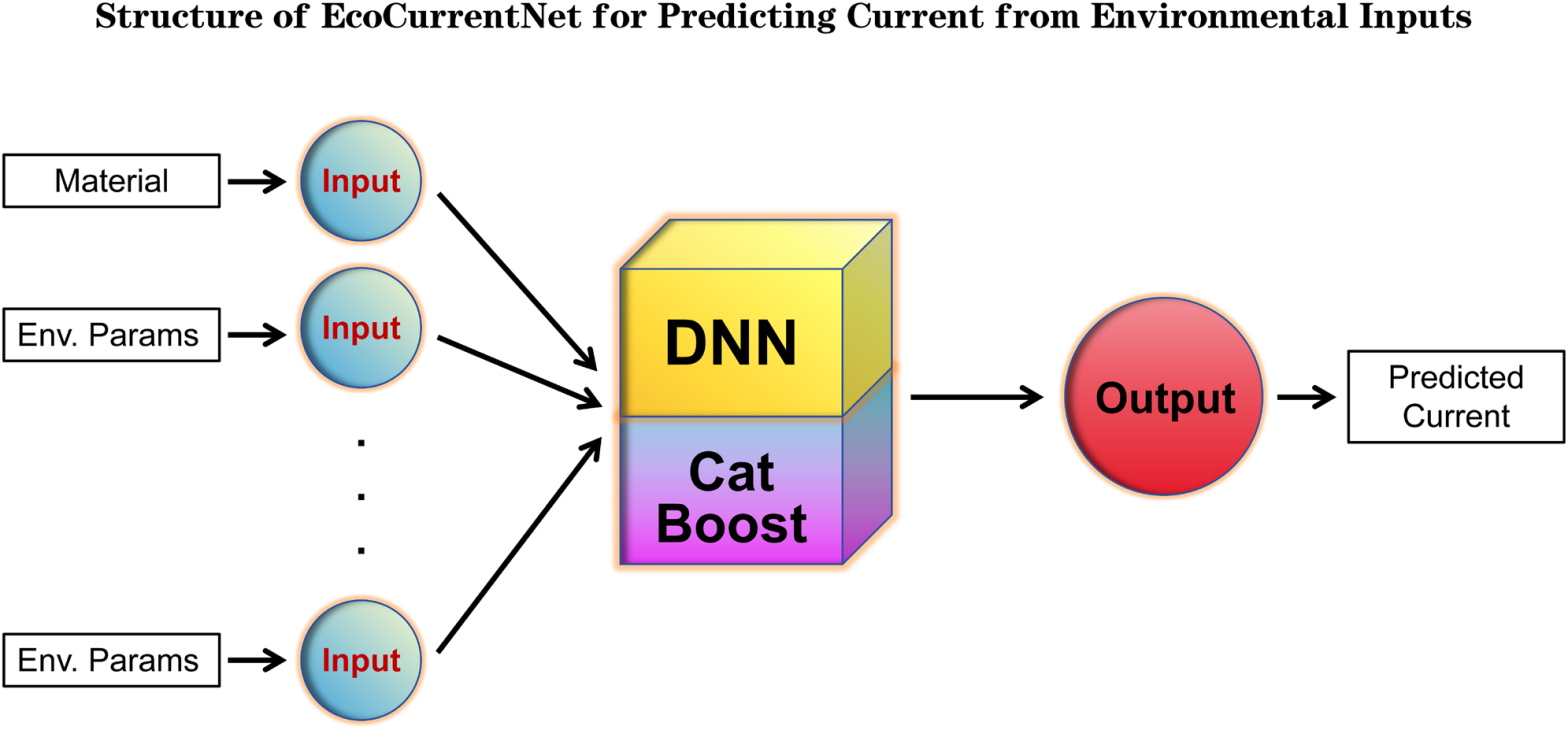
Architecture of EcoCurrentNet. This diagram illustrates how input parameters (such as light intensity, temperature, etc.) are processed through a DNN and integrated with a CatBoost regression layer, resulting in the final output predictions. The architecture combines the strengths of both DNN and CatBoost, enabling the model to handle complex feature interactions effectively and improve prediction accuracy.

### Model architecture

The flowchart in Fig. 5 illustrates the architecture of EcoCurrentNet, showing how input parameters such as light intensity, temperature, material type, and other factors are processed through a deep neural network (DNN) and integrated with a CatBoost regression layer. This hybrid architecture combines the strengths of both DNN and CatBoost, effectively handling complex feature interactions and improving prediction accuracy^33–35^.

**EcoCurrentNet_V4** is the highest-performing model in the **EcoCurrentNet series**, which consists of five different versions. From EcoCurrentNet_V1 to EcoCurrentNet_V5, the depth and complexity of the network gradually increase, with each version adding additional layers and more complex connections, enhancing the model’s ability to capture complex interactions between features. All models in the EcoCurrentNet series are specifically designed to predict the performance of optoelectronic materials under varying conditions. The EcoCurrentNet_V4 version integrates multiple convolutional layers and residual connections, among other advanced features, which enable it to capture the complex nonlinear relationships between input parameters and target responses more effectively than other versions. Due to its superior performance, EcoCurrentNet_V4 was selected as the preferred model for this research. EcoCurrentNet_V4 (from hereon referred to as EcoCurrentNet) strikes an ideal balance between network depth and computational efficiency, providing an architecture capable of handling complex multi-dimensional datasets while maintaining high accuracy and training stability.

The multiple convolutional layers of the model efficiently extract features from the input data, including parameters such as light intensity, temperature, material type, humidity, wavelength, and pressure. The convolutional layers are crucial for detecting local patterns and dependencies in the data, enabling the model to identify subtle yet important relationships between different material characteristics. The residual connections used in the architecture ensure training stability as the network depth increases, helping maintain gradient flow during backpropagation and alleviating the vanishing gradient problem that may arise in deep networks. These connections aid the model in learning complex relationships and improve the overall convergence of the network.

EcoCurrentNet’s architecture is inspired by advancements in deep learning, particularly in fields such as computer vision and natural language processing, where convolutional neural networks (CNNs) and residual connections have been proven effective in handling complex, high-dimensional data. The use of deep convolutional layers allows the model to automatically extract relevant features from multi-dimensional input data, a concept widely used in image recognition and sequence modeling tasks. Residual connections, which have gained significant attention in recent years, help mitigate the vanishing gradient problem, ensuring stable training in deeper networks. These design choices are particularly well-suited for modeling optoelectronic performance, where intricate dependencies between multiple material properties need to be captured and learned effectively.

The task of predicting optoelectronic material performance inherently involves nonlinear relationships between input parameters. For example, parameters such as temperature and humidity may affect material performance in highly nonlinear ways. EcoCurrentNet effectively captures these nonlinear relationships through its deep network architecture, offering higher prediction accuracy compared to simpler models. The deep structure allows it to detect complex patterns and interactions that might be overlooked in traditional linear regression or shallow neural networks.

**Fig. 6:**
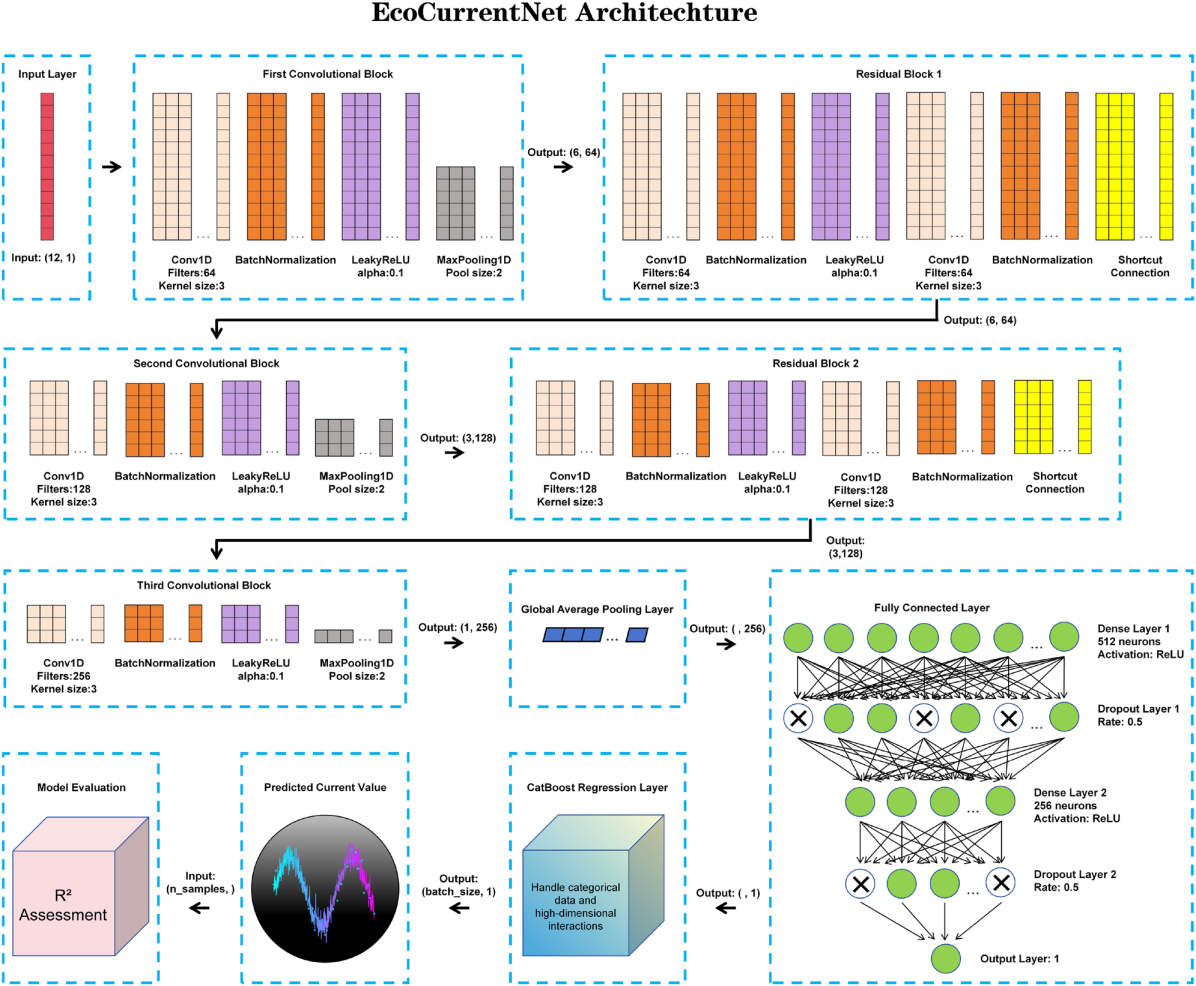
The EcoCurrentNet model consists of three convolutional blocks, each followed by a residual block. These convolutional blocks are responsible for extracting features from the optoelectronic materials. The residual blocks enhance the information flow by utilizing skip connections. After the convolutional and residual blocks, max-pooling layers are applied to downsample the features. Following these layers, fully connected layers process the features further. The output is then fed into the CatBoost regressor, which predicts the current. The model’s performance is evaluated using the R^2^ metric, with R^2^ values closer to 1 indicating better predictive performance.

The architecture of EcoCurrentNet is shown in Table 5 and Fig. 6, illustrating the integration of sequential convolutional layers and residual connections. Each convolutional layer is followed by an activation function (such as ReLU or Leaky ReLU) to introduce non-linearity, enhancing the model’s expressive power and enabling it to learn complex relationships between input features and output responses. The convolutional layers perform feature extraction on the input data through a sliding window, capturing local spatial or temporal dependencies. As the network depth increases, the convolutional layers progressively extract more abstract and higher-level features, improving the model’s ability to capture complex patterns.

Additionally, residual connections are introduced after each convolutional layer to effectively mitigate the vanishing gradient problem that may arise as the network depth increases. By directly passing the input to the next layer, the residual connections ensure the flow of important information, stabilizing the training process and facilitating the learning of deeper features. This enables EcoCurrentNet to better capture complex non-linear relationships in the data, especially in the interactions between multi-dimensional input parameters.

After the convolutional layers and residual connections, EcoCurrentNet incorporates fully connected layers (Dense Layer) to further integrate the high-level features extracted from the convolutional layers and map them to the final output space. In EcoCurrentNet, the first fully connected layer consists of 512 neurons and uses the ReLU activation function to introduce non-linearity. The output of this layer is passed through a subsequent Dropout layer to prevent overfitting and improve the model’s generalization ability. By progressively learning features from multiple layers, the fully connected layers capture more complex relationships, further enhancing the model’s predictive capabilities.

Finally, EcoCurrentNet integrates a CatBoost regression layer to enhance the model’s ability to handle high-dimensional, complex feature interactions. CatBoost excels in handling categorical data and missing values, capturing finer interactions between multiple features. Combined with the outputs from the deep neural network, the CatBoost regression layer further optimizes the model’s prediction accuracy. Its unique boosting mechanism allows the model to more precisely handle non-linear relationships, improving the overall prediction performance.

**Table 5:** EcoCurrentNet model architecture.

Layer type	Description	Details
Input Layer	Input data normalization	Multiple environmental and material parameters
Convolutional layer 1	Low-level feature extraction	64 filters, kernel size = 3
Convolutional layer 2	Complex feature extraction	128 filters, kernel size = 3
Convolutional lkayer 3	Deep feature extraction	256 filters, kernel size = 3
Activation function	Non-linear feature enhancement	LeakyReLU after each convolutional layer
Batch normalization	Stabilize training process	Applied after each convolutional layer
Residual connections	Enhance trainability	Skip connections between deeper layers
Global average pooling	Dimensionality reduction	Averages the output of each feature map
Fully connected layer 1	Feature combination	512 neurons, ReLU activation
Fully connected layer 2	Further feature compression	256 neurons, ReLU activation
Dropout	Prevent overfitting	Applied after each fully connected layer
Output layer	Final prediction	Single neuron, no activation function
CatBoost regression layer	Enhanced regression	CatBoost model for high-dimensional data

### Mathematical logic of EcoCurrentNet

In this section, we break down the mathematical logic of EcoCurrentNet, outlining the operations and transformations applied across its layers, including convolutional, residual connections, global average pooling, dense layers, and the CatBoost regression layer. Each operation is explained with its mathematical representation^36–38^.

**1. Input Layer** The input data to the model consists of a matrix $X$, where $n$ is the number of samples, and $m$ is the number of features. Each sample $\textbf{x}_i$ is a vector of features:

4 $$\begin{aligned} X = \begin{bmatrix} \textbf{x}_1 \\ \textbf{x}_2 \\ \vdots \\ \textbf{x}_n \end{bmatrix} \end{aligned}$$

*Where:* Each feature vector $\textbf{x}_i$ corresponds to a sample in the dataset^38,39^.

**2. Convolution Block** The convolution operation applies a filter $W^{(k)}$ of size $k$ to a sequence of input data $X$. The formula for the convolution operation is given as:

5 $$\begin{aligned} Z_i^{(k)} = \sum _{j=1}^{k} X_{i+j-1} \cdot W^{(k)}_j + b^{(k)} \end{aligned}$$

*Where:*

- $Z_i^{(k)}$ is the output of the convolution at position $i$.
- $W_j^{(k)}$ are the weights of the convolutional kernel at the $j$-th position.
- $X_{i+j-1}$ is the value from the input sequence at position $i + j - 1$, which is multiplied by the corresponding weight in the kernel.
- $b^{(k)}$ is the bias term added to the convolution result.

The operation involves sliding the kernel $W^{(k)}$ over the input sequence $X$ and calculating the weighted sum of input values for each region of the input sequence, with the bias term $b^{(k)}$ added to each output. This is a standard operation in convolutional layers, where the filter is applied to local regions of the input, and the weights $W^{(k)}_j$ are learned during training. In the case of EcoCurrentNet, this convolution operation plays a critical role in extracting spatial features from the input data, such as environmental factors and material properties, which are then used to predict optoelectronic material performance^39–41^.

**Batch Normalization and Leaky ReLU Activation** After the convolution operation, the output is passed through **Batch Normalization** followed by a **Leaky ReLU** activation function:

6 $$\begin{aligned} \textbf{Z}_i^{(k)} = \text {LeakyReLU}(\text {BatchNorm}(Z_i^{(k)})) \end{aligned}$$

**Batch normalization** is used to normalize the activations of the previous layer (in this case, the convolution output $Z_i^{(k)}$) by adjusting and scaling the activations. The formula for batch normalization is:

7 $$\begin{aligned} \hat{Z}_i^{(k)} = \frac{Z_i^{(k)} - \mu }{\sqrt{\sigma ^2 + \epsilon }} \end{aligned}$$

*Where:*

- $\mu$ is the mean of the batch.
- $\sigma ^2$ is the variance of the batch.
- $\epsilon$ is a small constant added for numerical stability.

This normalization helps improve convergence speed during training and reduces the sensitivity of the model to the initial weight values, enabling faster training. In EcoCurrentNet, batch normalization ensures that the feature maps produced by the convolution layers are well-scaled and centered, aiding in the stability and efficiency of the training process^42^.

**Leaky ReLU Activation** is a variant of the standard ReLU activation function, which allows for small negative values when the input is less than zero. The formula for Leaky ReLU is:

8 $$\begin{aligned} \text {LeakyReLU}(x) = \max (\alpha x, x) \end{aligned}$$

*Where:*

- $\alpha$ is a small constant (often set to 0.01) that controls the slope for negative values.

Leaky ReLU introduces non-linearity to the network while ensuring that some gradient flows even for negative inputs, helping mitigate the problem of “dying ReLUs,” where neurons stop learning if they output zeros for all inputs. This is particularly relevant in EcoCurrentNet, as it ensures that the network maintains the capacity to learn complex patterns in the data, even when some neurons exhibit low activations^43–45^.

Together, these operations-convolution, batch normalization, and Leaky ReLU-enable the network to learn complex features from the input data while stabilizing activations and improving training efficiency. The convolution operation captures local patterns in the input, while batch normalization and Leaky ReLU facilitate faster and more stable learning. In EcoCurrentNet, these operations work synergistically to process environmental and material data efficiently, contributing to the model’s overall performance in predicting optoelectronic material behavior under varying conditions^36–38^.

**3. Residual Block**

A **residual block** facilitates the learning of identity mappings, which aids in training deeper networks by mitigating the vanishing gradient problem. It consists of two convolutional layers and a **skip connection** (or **shortcut**) that directly adds the input of the block to its output. This design enables the network to learn residual functions, simplifying the optimization of deeper architectures, as described by He et al.^46^.

The mathematical representation of the residual block is as follows:

9 $$\begin{aligned} \textbf{Z}_i^{\text {res}} = \text {ReLU} \left( \sum _{j=1}^{k} \left( \sum _{l=1}^{m} X_{i+l} \cdot W^{(k)}_j + b^{(k)} \right) + \textbf{Z}_i \right) \end{aligned}$$

Here, the variables are defined as:

- $X_{i+l}$ denotes the input at the ${l}$-th position of the convolutional kernel at layer $k$.
- $W_j^{(k)}$ is the weight matrix of the kernel at position $j$.
- $b^{(k)}$ represents the bias term added to the convolution output.
- The first summation represents the convolution operation applied across the input $X$, across positions $i$ to $i+m$.
- The residual connection adds the input $\textbf{Z}_i$ from the previous layer directly to the convolutional output, allowing the network to learn the difference between input and output.

**Residual Learning Framework**

Residual learning enables the network to model the residual function $F(X) = H(X) - X$, where $H(X)$ is the desired output. Instead of learning the direct mapping $H(X)$, the network learns the difference, $F(X)$, which simplifies training for deeper networks^47,48^.

The operation can be expressed as:

10 $$\begin{aligned} \textbf{Z}_i^{\text {res}} = F(X) + X \end{aligned}$$

*where:*

- $F(X)$ is the residual function that represents the difference between the desired output $H(X)$ and the input $X$.
- $X$ is the input to the residual block, added back to $F(X)$ to form the final output.

This approach helps mitigate training difficulties, such as vanishing or exploding gradients, which often arise in deeper networks^47^.

**Activation and output**

After the convolution and residual addition, the output is passed through a **ReLU** activation function to introduce non-linearity^49^. The final output is then given by:

11 $$\begin{aligned} \textbf{Z}_i^{\text {res}} = \text {ReLU}(F(X) + X) \end{aligned}$$

The ReLU activation ensures the network can model complex, non-linear functions, while the skip connection helps prevent the vanishing gradient problem by facilitating gradient flow across layers^42^.

**Gradient propagation and backpropagation**

During backpropagation, the gradient of the loss function $\mathcal {L}$ with respect to the input $X$ is computed. The residual connection ensures that the gradient does not diminish across many layers^39^. The gradient with respect to $X$ is:

12 $$\begin{aligned} \frac{\partial \mathcal {L}}{\partial X} = \frac{\partial \mathcal {L}}{\partial \textbf{Z}_i^{\text {res}}} \cdot \frac{\partial \textbf{Z}_i^{\text {res}}}{\partial X} \end{aligned}$$

Since the residual block includes the direct addition of $X$, the gradient with respect to $X$ is propagated efficiently, even across deep networks.

This approach enables EcoCurrentNet to achieve more stable and efficient training, ensuring that the model learns complex relationships in optoelectronic material performance without being hindered by the challenges of deep network training^36,41^. The use of residual blocks allows EcoCurrentNet to maintain high performance while processing intricate data sets, facilitating faster convergence and more accurate predictions.

**Advantages of residual blocks in EcoCurrentNet**

The inclusion of residual blocks in EcoCurrentNet provides several key advantages. By enabling the network to learn residual functions, it improves the flow of gradients, which is particularly beneficial when dealing with complex optoelectronic data. The skip connections help mitigate the vanishing gradient problem, allowing EcoCurrentNet to be trained effectively even with deeper architectures. This, in turn, contributes to enhanced model accuracy and robustness, crucial for making reliable predictions in the challenging domain of optoelectronic material performance^40,44^.

**4. Global average pooling (GAP)** After several convolutional and residual layers, the feature maps are passed through a Global Average Pooling (GAP) layer. GAP is a downsampling technique that reduces each feature map to a single scalar value, thereby capturing global spatial information. The operation can be mathematically expressed as:

13 $$\begin{aligned} \textbf{Z}_i^{\text {GAP}} = \frac{1}{n} \sum _{j=1}^{n} Z_j^{\text {res}} \end{aligned}$$

*Where:*

- $Z_j^{\text {res}}$ represents the feature maps obtained after the residual blocks.
- $n$ is the number of spatial positions, corresponding to the length of the sequence or the size of the feature map.

In this operation, the sum is taken over all spatial positions of each feature map. By averaging the feature map values, GAP creates a compact and invariant representation of the input, which is particularly useful for capturing global features across the spatial dimensions. This transformation reduces the high-dimensional data into a lower-dimensional vector, while still retaining the most significant information for downstream processing^40,46,50^.

Mathematically, GAP can be seen as a form of dimensionality reduction that retains the essence of the learned features. This not only aids in reducing computational complexity but also prevents overfitting by forcing the model to focus on the global characteristics of the data. In the context of EcoCurrentNet, this operation effectively distills the spatially encoded environmental and material information, providing a condensed representation that aids in predicting optoelectronic material behavior efficiently^36,51^.

Furthermore, by eliminating the need for fully connected layers, GAP contributes to the model’s simplicity and scalability, as the output size is independent of the input size, making it particularly suited for handling varying input lengths^42^.

**5. Dense (Fully connected) layers** The output from the GAP layer is passed through fully connected layers. Let $Z_i^{\text {GAP}}$ be the input to the dense layer, and let $W^{\text {dense}}$ be the weight matrix for the dense layer, with biases $b^{\text {dense}}$. The output of the dense layer is given by:

14 $$\begin{aligned} \textbf{Z}_i^{\text {dense}} = \sum _{j=1}^{n} \textbf{Z}_j^{\text {GAP}} \cdot W^{\text {dense}}_i + b^{\text {dense}}_i \end{aligned}$$

*Where:*

- $\textbf{Z}_i^{\text {GAP}}$ is the $i$-th feature vector output from the Global Average Pooling (GAP) layer.
- $W^{\text {dense}}_i$ is the weight associated with the $i$-th node in the dense layer.
- $b^{\text {dense}}_i$ is the bias term for the $i$-th node in the dense layer.
- The summation $\sum _{j=1}^{n} \textbf{Z}_j^{\text {GAP}} \cdot W^{\text {dense}}_i$ is the dot product between the input feature vector $\textbf{Z}_j^{\text {GAP}}$ and the weight matrix $W^{\text {dense}}_i$.

The result is then passed through a ReLU activation:

15 $$\begin{aligned} \textbf{Z}_i^{\text {dense-out}} = \text {ReLU}(\textbf{Z}_i^{\text {dense}}) \end{aligned}$$

*Where:*

- $\textbf{Z}_i^{\text {dense}}$ is the linear transformation output before the ReLU activation.
- ReLU is a non-linear activation function defined as $\text {ReLU}(x) = \max (0, x)$, which introduces non-linearity into the network and helps to avoid vanishing gradients^49^.

Dense layers play a crucial role in deep learning architectures by mapping extracted features into a higher-dimensional space and facilitating the final prediction. These layers consolidate and transform the output from earlier stages into a format suitable for downstream tasks^41^. The application of fully connected layers, combined with non-linear activations like ReLU, is essential for enabling deep networks to learn complex feature interactions effectively^52^.

In EcoCurrentNet, the dense layers refine the learned features from previous layers, enhancing the model’s ability to predict optoelectronic material behavior efficiently. The use of ReLU ensures sparsity in the network, mitigating issues like vanishing gradients and improving training stability^42^. This process involves a linear transformation (dot product with weights) followed by a non-linear activation, which maps the extracted features to a higher-dimensional space, preparing them for the final prediction. The final output layer is tailored to the specific task, whether regression or classification.

**6. CatBoost regression layer** The final step in EcoCurrentNet is the **CatBoost regression layer**, which applies gradient-boosted decision trees as an ensemble method. CatBoost iteratively refines the predictions by fitting decision trees to the residual errors of previous predictions, enhancing the model’s ability to capture non-linear relationships in the data. The output $\textbf{Z}_i^{\text {dense-out}}$ from the dense layers is used as input for the CatBoost regressor, which refines the prediction:

16 $$\begin{aligned} y = \text {CatBoost}(\textbf{Z}_i^{\text {dense-out}}) \end{aligned}$$

*Where:*

- $y$ is the predicted output (e.g., material property or performance measure).
- $\textbf{Z}_i^{\text {dense-out}}$ is the feature vector from the dense layers, used as input to the CatBoost regressor.

The learning process involves minimizing residuals through an iterative boosting process. At each iteration $t$, the residual error is computed as the difference between the true target value and the predicted value from the previous iteration:

17 $$\begin{aligned} r_t = y_{\text {true}} - y_{\text {predicted}}^{(t-1)} \end{aligned}$$

*Where:*

- $r_t$ is the residual error at iteration $t$, which represents the difference between the true target value and the predicted value from the previous iteration.
- $y_{\text {true}}$ is the actual target value (true value) for the given data point.
- $y_{\text {predicted}}^{(t-1)}$ is the predicted value from the previous iteration, which is updated at each boosting iteration to minimize the residual error.

The decision trees are trained to minimize these residuals, enabling the model to improve its approximation of non-linear patterns. This iterative process continues until the predictions converge, gradually improving the model’s performance. The CatBoost model’s ability to handle categorical features effectively is one of its key advantages^7,53,54^. Additionally, gradient boosting methods like CatBoost have been extensively reviewed for their efficiency in large-scale datasets^55^.

In the context of regression models, CatBoost has been shown to effectively handle categorical data and reduce bias, making it an ideal choice for modeling complex relationships in materials science applications^7,53^. Additionally, it has been demonstrated to outperform many other gradient boosting algorithms in terms of efficiency and predictive accuracy, especially when dealing with datasets containing categorical variables^55^. The iterative nature of gradient boosting allows for robust predictions, minimizing errors such as residuals and improving model performance iteratively^56^. Moreover, gradient boosting models like CatBoost are effective in refining the prediction by learning from the residuals, as discussed in literature related to boosting methods and regression performance metrics^57,58^.

The performance of EcoCurrentNet is evaluated using the $R^2$ score, which quantifies how well the model’s predictions explain the variance in the target data. The formula for $R^2$ is:

18 $$\begin{aligned} R^2 = 1 - \frac{\sum _{i=1}^{N} (y_{\text {true}, i} - y_{\text {pred}, i})^2}{\sum _{i=1}^{N} (y_{\text {true}, i} - \bar{y}_{\text {true}})^2} \end{aligned}$$

*Where:*

- $N$ is the number of samples in the dataset.
- $y_{\text {true}, i}$ is the true target value for the $i$-th sample.
- $y_{\text {pred}, i}$ is the predicted value for the $i$-th sample.
- $\bar{y}_{\text {true}}$ is the mean of the true target values.

A higher $R^2$ score indicates that the model’s predictions are more consistent with the true values, with $R^2$ closer to 1 indicating better model performance.

The prediction process in EcoCurrentNet is refined through the iterative boosting process in CatBoost. The prediction for each iteration $t$ is updated by adding the contribution of the current decision tree:

19 $$\begin{aligned} y_{\text {pred}}^{(t)} = y_{\text {pred}}^{(t-1)} + \eta \cdot f_t(\textbf{Z}_i^{\text {dense-out}}) \end{aligned}$$

*Where:*

- $f_t(\textbf{Z}_i^{\text {dense-out}})$ is the decision tree trained at iteration $t$.
- $\eta$ is the learning rate that controls the contribution of each tree.
- $y_{\text {pred}}^{(t)}$ is the updated prediction after iteration $t$.

The overall prediction is the sum of all decision trees’ contributions:

20 $$\begin{aligned} y = \sum _{t=1}^{T} f_t(\textbf{Z}_i^{\text {dense-out}}) \end{aligned}$$

*Where:*

- $T$ is the total number of iterations (trees) in the CatBoost model.

Thus, the CatBoost regression layer in EcoCurrentNet plays a vital role in refining predictions by utilizing gradient boosting, improving the model’s ability to capture complex, non-linear relationships while using $R^2$ as the key metric for assessing model performance.

**Summary** EcoCurrentNet integrates 1D convolutions, residual connections, and a CatBoost regression layer to process and predict optoelectronic material properties under varying conditions. The convolutions extract local patterns, the residual blocks ensure stable learning across deeper layers, and the CatBoost regressor refines predictions by learning complex, non-linear relationships between features. The entire model can be expressed mathematically as follows:

21 $$\begin{aligned} y_{\text {pred}} = \text {CatBoost}\left( \text {Dense} \left( \text {MaxPooling} \left( \text {Residual}\left( \text {Conv1D}_3 + \text {Residual}(\text {Conv1D}_2) + \text {Conv1D}_1 \right) \right) \right) \right) \end{aligned}$$

*Where:*

- $\text {Conv1D}_1, \text {Conv1D}_2, \text {Conv1D}_3$ are the 1D convolutional layers that extract hierarchical features from the input data at different levels.
- $\text {Residual}(\text {Conv1D}_2)$ and $\text {Residual}(\text {Conv1D}_3)$ represent the residual connections that help the gradient flow through deeper layers and prevent vanishing gradients during backpropagation.
- $\text {MaxPooling}$ is the max pooling layer that reduces the dimensionality of the feature map and retains the most important features.
- $\text {Dense}$ refers to the fully connected layers that refine the learned features from the previous layers.
- $\text {CatBoost}$ is the gradient-boosted decision tree model applied to the final output layer to learn non-linear relationships and refine the model’s predictions.

EcoCurrentNet was developed to enhance the prediction of optoelectronic material properties by combining deep learning and decision tree-based boosting. Its unique combination of convolutional layers, residual connections, and CatBoost regression offers several advantages for this domain, including improved accuracy and efficiency in predicting material performance under variable environmental conditions. By integrating these methods, EcoCurrentNet bridges gaps in existing prediction models that often struggle to capture complex, non-linear relationships. This model advances the field of material science by offering a more reliable tool for performance prediction, thereby enabling better material design and optimization for real-world applications.

### Training and validation and evaluation metrics

The design of the training and evaluation process plays a crucial role in ensuring the reliability and effectiveness of EcoCurrentNet. Properly structured training phases enable the model to learn complex patterns in the data, while the validation process provides continuous feedback to guide optimization and prevent overfitting. Evaluation metrics, such as R^2^, are essential for assessing the model’s performance during both training and validation. This metric offers a comprehensive view of the model’s predictive accuracy and generalization ability, helping to fine-tune the model and confirm its robustness. By carefully selecting and monitoring these metrics, **this research** ensures that the final model not only performs well on training data but also generalizes effectively to unseen data, making it a reliable tool for optoelectronic material predictions.

**1. Training** The training process of EcoCurrentNet is carefully designed to ensure both strong initial learning and fine-tuned optimization. The model is first trained using a feedforward neural network, followed by fine-tuning to improve prediction accuracy and generalization ability. In the initial training phase, the model learns the main patterns in the data, while in the fine-tuning phase, the model’s parameters are further optimized to improve its performance on the validation set. Through this approach, **this research** progressively enhances its performance over two training rounds, ultimately achieving high predictive capability.

**Fig. 7:**
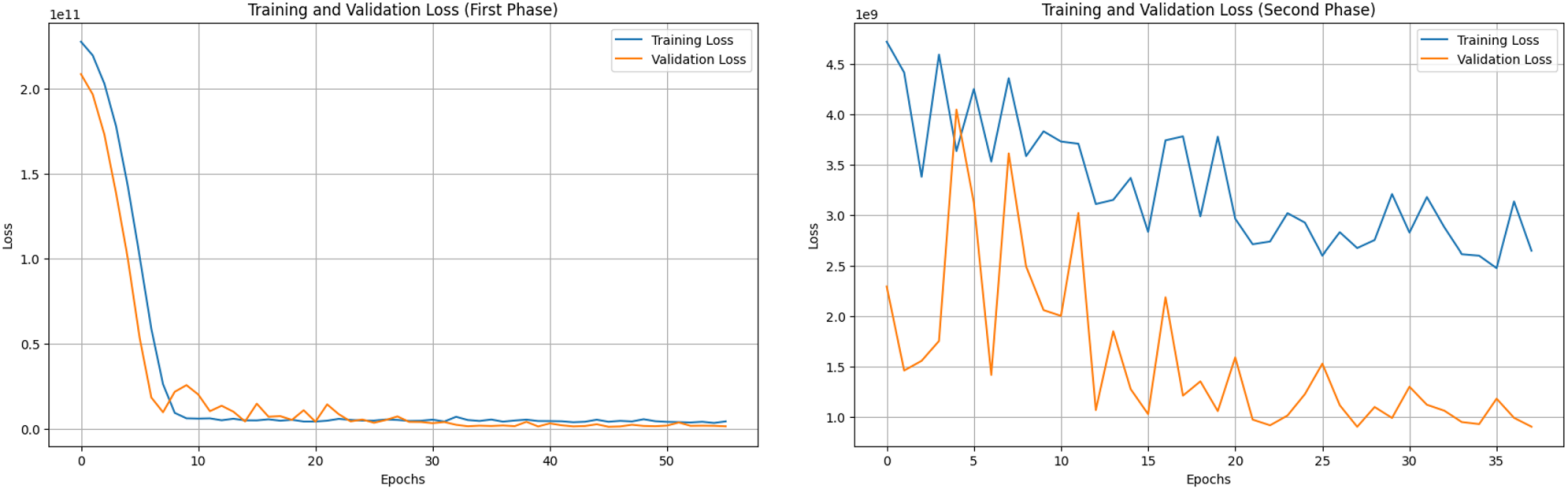
Training and validation loss during the two training phases of EcoCurrentNet. The first phase uses the RMSprop optimizer, while the second phase introduces dynamic learning rate adjustments for further optimization.

**Training phases and optimization process** EcoCurrentNet undergoes training in two phases, each aimed at refining the model’s performance. The first phase focuses on initial training using the RMSprop optimizer, while the second phase continues with the ReduceLROnPlateau technique for further optimization.

In the first phase, the model is trained using the RMSprop optimizer, with early stopping applied to prevent overfitting. The validation loss is monitored to select the optimal training epoch. This approach helps avoid overtraining and ensures that the model maintains good generalization ability. The continuous improvement in validation loss indicates that the model is effectively fitting the training data and maintaining strong performance on the validation data, which aligns with the findings of previous studies on deep learning optimization strategies^59^.

In the second phase, the model undergoes additional training with the ReduceLROnPlateau technique, which dynamically adjusts the learning rate. This enables finer tuning as the model approaches the optimal solution, improving both training stability and precision. The technique facilitates more refined adjustments as the model nears convergence, further optimizing its performance, in line with the optimization strategies discussed by Thakur et al. (see Fig. 7)^60^.

**Training and inference time** The training process for EcoCurrentNet, using 10,000 samples and 100 epochs, required approximately 45 seconds in total on a standard workstation equipped with an Intel i7 CPU and 32GB RAM. This includes both Phase 1 and Phase 2 training, made possible by early stopping that halts training once the validation loss fails to improve for 10 consecutive epochs. Inference for a single sample takes less than 5 milliseconds, enabling deployment in real-time or embedded scenarios.

Compared to traditional machine learning models, EcoCurrentNet’s training time is moderately higher due to the DNN component. However, its inference time remains competitive, especially when executed in batch mode. These findings demonstrate that EcoCurrentNet is not only accurate but also computationally efficient, making it well-suited for practical applications involving large-scale environmental data.

**Ensuring model robustness and generalization ability** Additionally, the ModelCheckpoint callback function is used to automatically save the model’s best weights based on validation loss, ensuring that the final model is the one with the best performance. This strategy effectively alleviates overfitting and ensures that the model can maintain strong predictive power and adaptability when faced with unseen data.

These well-designed training strategies not only enhance the model’s prediction accuracy but also ensure its robustness and reliability in complex tasks. With a scientifically planned training scheme, EcoCurrentNet has become an advanced tool for efficiently handling optoelectronic material prediction tasks, standing out in the field for its precision and superior generalization ability^61^.

**2. Evaluation metrics** In this study, $R^2$ (coefficient of determination) was chosen as the primary evaluation metric for the performance of the EcoCurrentNet model. The choice of $R^2$ is closely related to the specific objectives of the research and the characteristics of the model:

**Suitability for regression tasks** Since this study involves predicting the performance of optoelectronic materials, the goal is to capture the complex relationships between input features (such as physical and environmental properties of materials) and output performance. As one of the most commonly used evaluation metrics in regression problems, $R^2$ measures the model’s ability to explain the variance in the data. Particularly when dealing with high-dimensional feature data, $R^2$ provides a clear quantifiable measure of the model’s ability to capture the complex, nonlinear relationships between input variables.

**Capturing Complex Nonlinear Relationships** EcoCurrentNet combines deep neural networks and CatBoost models, which possess strong nonlinear modeling capabilities. $R^2$ is an ideal evaluation metric because it reflects not only the model’s fit for linear relationships but also its ability to capture underlying nonlinear relationships in the data. In tasks where multiple variables and their interactions must be considered simultaneously, $R^2$ provides a global evaluation metric that helps assess the model’s overall goodness of fit.

**Model generalization ability** While other metrics, such as MAE or MSE, reflect the absolute level of error, they may not directly indicate the model’s global fit in high-dimensional feature spaces. In contrast, $R^2$ effectively reflects the model’s prediction ability on new data by comparing its performance to a baseline model (e.g., a simple mean-prediction model). In this task, using $R^2$ allows for a clear demonstration of EcoCurrentNet’s overall predictive capability and robustness, especially in predicting optoelectronic material performance under complex and varying conditions.

**Fair comparison across models** In this study, the performance of EcoCurrentNet was compared with various traditional regression models (such as Ridge Regression, Random Forest, Decision Trees, etc.). Since different models have different characteristics and complexities, $R^2$ provides a standardized metric that allows the performance of these models to be compared effectively under the same benchmark. In this context, $R^2$ serves as a fair and consistent evaluation standard that helps determine the advantages of EcoCurrentNet relative to other models.

**Fig. 8:**
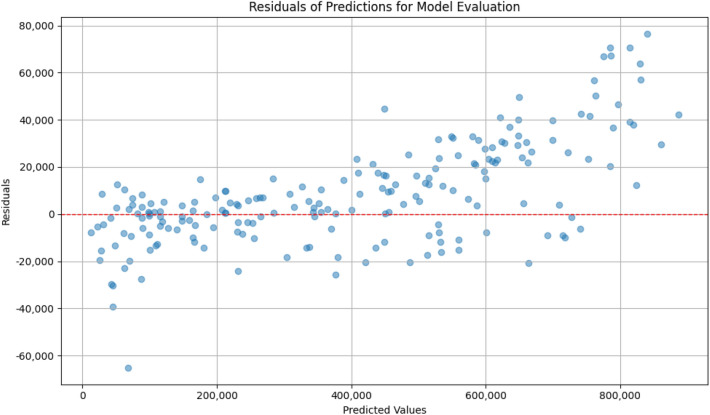
The plot shows the distribution of residuals (differences between the predicted and true values) for model evaluation. Ideally, residuals should be randomly distributed around zero, indicating that the model is not biased and has captured the underlying data patterns effectively.

To further evaluate the model’s predictive performance, the residuals and the true versus predicted values are examined. The residuals plot helps assess how well the model captures the underlying patterns in the data, revealing any systematic deviations from the model’s predictions. As shown in Fig. 8, the residuals are randomly distributed around zero, indicating that the model’s errors are unbiased and not exhibiting any obvious patterns. This suggests that EcoCurrentNet generalizes well and is not overfitting the training data.

**Fig. 9:**
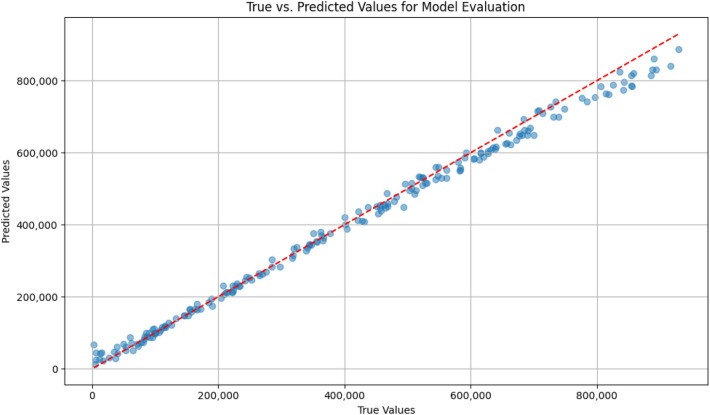
This scatter plot compares the true values of the optoelectronic material performance against the predicted values from EcoCurrentNet. A close alignment between the two sets of values indicates strong predictive accuracy and generalization ability, with the model effectively capturing the underlying relationships in the data.

Additionally, the true versus predicted values plot (see Fig. 9) visually demonstrates the alignment between the model’s predictions and the actual observed values. The close correlation between these values further supports the model’s high accuracy, with $R^2 = 0.996526$, showcasing the model’s ability to capture the complex nonlinear relationships between input features and output performance.

**3. Consistency with theoretical behavior**

In addition to evaluating the predictive performance of EcoCurrentNet, we further validated its effectiveness by comparing its outputs with established theoretical behavior. Specifically, we investigated how EcoCurrentNet can predict the electrical performance of optoelectronic materials-such as current-based on learned environmental and physical parameters, thereby indirectly capturing characteristics associated with current–voltage (I–V) behavior. This process is crucial for modeling material performance under varying environmental conditions^62,63^.

**Theoretical framework** The behavior of optoelectronic materials is commonly described using the Shockley diode equation, which expresses current (*I*) as a function of voltage (*V*):

22 $$\begin{aligned} I = I_s \left( e^{\frac{qV}{kT}} - 1 \right) \end{aligned}$$

where:

- $I$ is the current,
- $I_s$ is the saturation current,
- $q$ is the charge of the electron,
- $V$ is the applied voltage,
- $k$ is the Boltzmann constant,
- $T$ is the temperature (in Kelvin).

The equation describes an exponential relationship between current and voltage, forming the theoretical foundation for many optoelectronic devices.

**Comparison with theoretical predictions** To assess whether EcoCurrentNet can effectively model optoelectronic material behavior, we used the Shockley diode equation to simulate the I-V characteristics of silicon-based materials and compared the results with predictions made by EcoCurrentNet. In our experimental setup, key variables such as bias voltage, temperature, and humidity were carefully controlled to ensure reproducibility. The comparison shows that the model’s predictions deviate within a margin of $\pm 2.5\%$ from the theoretical curves, indicating that EcoCurrentNet successfully captures the essential physical principles underlying the materials’ behavior under different environmental conditions^35,64,65^.

In addition to silicon, we conducted similar comparisons on other materials (e.g., GaAs, Ge, InP), which demonstrated comparable predictive accuracy. These results further support the generalizability and effectiveness of EcoCurrentNet across a variety of materials and experimental conditions.

**Controlled variables and laboratory constraints** To ensure the reliability and validity of the predictions, we controlled multiple experimental variables. In particular, we regulated bias voltage, temperature, and humidity, which are critical factors affecting optoelectronic material performance. Due to limitations of the laboratory environment, we were unable to control additional variables such as surface condition and illumination intensity. These uncontrolled factors may have some influence on the final model performance, which represents a direction for future research and refinement^66–68^.

**Significance of the validation** The comparison with theoretical behavior reinforces the predictive accuracy and reliability of EcoCurrentNet. This validation highlights the model’s ability not only to capture fundamental physical relationships but also to adapt to the complexity of real-world environmental conditions.

Through these comparisons, we demonstrate that EcoCurrentNet is a robust and trustworthy tool for predicting the performance of optoelectronic materials, offering valuable support for both theoretical research and practical applications in the field^29^.

## Conclusion

This study presents EcoCurrentNet, an advanced hybrid modeling framework that effectively integrates deep neural networks with CatBoost regression for predicting the performance of optoelectronic materials under a wide range of environmental conditions. The model demonstrates high predictive accuracy, achieving an R^2^ value of 0.9847 on the test set, and effectively captures complex feature interactions between both categorical and continuous input parameters.

A key innovation of this study is the integration of twelve environmental factors into the model. These factors include common variables such as light intensity, temperature, and humidity, along with other critical yet subtle variables like material density, surface treatment, impurity concentration, and electric field strength. Although these additional factors might show weaker correlations in controlled lab conditions, they are essential for accurate prediction in real-world applications, such as high-altitude or high-humidity environments. Surface treatments and material density, for example, have a direct and significant impact on the performance of optoelectronic materials, particularly under environmental conditions that cannot be easily replicated in laboratory settings. Including these factors ensures that the model simulates real-world scenarios more comprehensively, providing more reliable predictions of material performance in diverse conditions^69–71^.

The model’s predictions align with theoretical expectations, as demonstrated by simplified test cases. For example, as light intensity and electric field strength increase, the predicted current increases linearly, which is consistent with the classical drift-diffusion model. Temperature variations lead to an increase in current, which saturates at higher temperatures, mirroring behavior observed in semiconductor materials.

Furthermore, EcoCurrentNet’s efficiency was validated through training and inference time tests. Training the model on an Intel i7 system with 32GB of RAM took less than 45 seconds, with inference predictions occurring in milliseconds, demonstrating the model’s practical utility for real-time applications^72–76^.

The ability of EcoCurrentNet to integrate a broad range of environmental variables makes it particularly useful for applications in extreme or unconventional environments, such as aerospace, high-altitude, extreme temperature, and high-pressure conditions, where optoelectronic materials must perform reliably. By providing accurate predictions of optoelectronic material performance in these environments, EcoCurrentNet contributes to the optimization and design of materials for high-risk, real-world applications.

Future research will focus on validating EcoCurrentNet with experimental data and applying it to other types of functional materials, further expanding its relevance and utility in a broader range of industries and environmental conditions^77–81^.

### Future directions: expanding the potential and applications of EcoCurrentNet

This study highlights the significant advancements EcoCurrentNet offers in predicting the performance of optoelectronic materials, particularly in capturing complex, multi-variable nonlinear interactions. By leveraging deep learning and machine learning, this research not only enhances practical applications in material design but also contributes to the theoretical framework of material science, offering novel insights into the behavior of optoelectronic materials under varying conditions. Moving forward, the model’s capabilities can be further developed and extended in the following ways:

1. **Incorporation of specific and subtle environmental factors:** Enhancing EcoCurrentNet to include both common (e.g., temperature, light intensity) and subtle but impactful variables (e.g., pressure, material density, surface treatment) ensures predictive accuracy in challenging real-world conditions. This allows the model to simulate realistic scenarios where variable interactions are nonlinear and not easily isolated in laboratory environments.
2. **Multi-scale modeling:** Integrating micro-scale properties, including crystal structures and defect states, into the model can enable a comprehensive understanding of how environmental factors influence material performance at different scales. Multi-scale material design using machine learning is an active area of research, which can further strengthen the model’s predictive power ^24,25^.
3. **Adaptive real-time learning:** Implementing online or incremental learning approaches can allow EcoCurrentNet to adapt its prediction strategies dynamically in response to changing environmental inputs, ensuring continuous high accuracy and efficiency. Such adaptive strategies are important for accelerating material discovery and have been incorporated into recent machine learning models ^82,83^.
4. **Interdisciplinary applications:** The EcoCurrentNet framework has potential beyond optoelectronics, with applications extending into renewable energy, climate monitoring, and smart manufacturing. This highlights EcoCurrentNet’s versatility as a tool for complex predictive tasks across various scientific fields ^11,83^.
5. **Automated discovery of new variables:** Leveraging EcoCurrentNet’s feature extraction capability could help identify previously unconsidered variables from data, such as novel environmental factors or microstructural characteristics. This approach, which has shown promise in materials discovery, can significantly enhance the model’s accuracy and comprehensiveness ^24,82^.
6. **High-dimensional data integration:** The model can be expanded to integrate data from various sensor inputs and experimental setups, enabling the exploration of new variables that impact material properties. The integration of high-dimensional data is key for improving the predictive capabilities of machine learning models in material science ^11,84^.
7. **Data augmentation and generative approaches:** Using data augmentation techniques to extend training datasets can reveal hidden variables and ensure robust training across diverse scenarios, improving the model’s ability to identify previously overlooked factors. These approaches have been effective in enhancing the robustness and generalizability of models ^82,83^.
8. **Cross-disciplinary exploration:** Insights from materials science, physics, and environmental studies can guide EcoCurrentNet to uncover new predictive variables not captured by traditional methods, boosting its performance through interdisciplinary collaboration ^11,25^.
9. **Reinforcement learning for adaptive strategy:** Integrating reinforcement learning frameworks allows EcoCurrentNet to optimize its variable selection and prediction strategies over time. Real-time monitoring and feedback will enable the model to autonomously adapt and include new variables, ensuring long-term reliability and adaptability ^24,82^.
10. **Robust prediction under extreme and uncontrolled conditions:** Future versions of EcoCurrentNet could be further optimized for performance in extreme environments, such as outer space, deep sea, polar regions, high-altitude plateaus, or high-radiation areas, where physical testing is challenging. By refining its ability to simulate and generalize across such conditions using diverse environmental variables, EcoCurrentNet can become a valuable predictive tool for mission-critical applications in aerospace, defense, and remote sensing technologies^85–88^.

These prospective directions underscore EcoCurrentNet’s potential as a comprehensive tool for advancing future material design and predictive analytics. By incorporating both dominant and subtle environmental parameters, the model extends its relevance to mission-critical applications under extreme and variable conditions, enabling materials research to address real-world complexities with theoretical rigor and practical utility.

## Data Availability

The datasets generated during this study are available in the GitHub repository: https://github.com/Alvy2024/Optoelectronic-current-simulation.git. For direct access to the dataset file: generated_data.xlsx.

## Code availability

The code used for the simulations and analysis in this study is publicly available at the GitHub repository: https://github.com/Alvy2024/Optoelectronic-current-simulation.git.
